# Molecular mechanism of ferroptosis and its role in the occurrence and treatment of diabetes

**DOI:** 10.3389/fgene.2022.1018829

**Published:** 2022-09-09

**Authors:** Guanghui Du, Qi Zhang, Xiaobo Huang, Yi Wang

**Affiliations:** ^1^ Department of Outpatient, Sichuan Academy of Medical Science and Sichuan Provincial People’s Hospital, University of Electronic Science and Technology of China, Chengdu, China; ^2^ School of Medicine, University of Electronics and Technology of China, Chengdu, China; ^3^ Department of Critical Care Medicine, Sichuan Academy of Medical Science and Sichuan Provincial People’s Hospital, University of Electronic Science and Technology of China, Chengdu, China

**Keywords:** ferroptosis, diabetes, ROS, GPX4, lipid peroxide, ferroptosis inhibitor

## Abstract

Ferroptosis is an iron-dependent programmed cell death, which is different from apoptosis, necrosis, and autophagy. Specifically, under the action of divalent iron or ester oxygenase, unsaturated fatty acids that are highly expressed on the cell membrane are catalyzed to produce lipid peroxidation, which induces cell death. In addition, the expression of the antioxidant system [glutathione (GSH) and glutathione peroxidase 4 (GPX4)] is decreased. Ferroptosis plays an important role in the development of diabetes mellitus and its complications. In this article, we review the molecular mechanism of ferroptosis in the development of diabetes mellitus and its complications. We also summarize the emerging questions in this particular area of research, some of which remain unanswered. Overall, this is a comprehensive review focusing on ferroptosis-mediated diabetes and providing novel insights in the treatment of diabetes from the perspective of ferroptosis.

## Introduction

In 2012, Brent R. Stockwell and his team found that erastin, a selectively lethal small molecule of oncogenic RAS, triggered a unique form of iron-dependent nonapoptotic cell death—a new form of cell death, called ferroptosis (Dixon et al., 2012). Ferroptosis is a kind of regulated cell death that primarily relies on iron-mediated oxidative damage and follows cell membrane damage (Chen et al., 2021a). Unlike previous forms of cell death, cells that experience ferroptosis exhibit atypical features of apoptosis. Morphologically, in erastin-induced cell death, mitochondria become smaller with increasing membrane density and the number of mitochondrial cristae decreases, without swelling of the cytoplasm and organelles (Lei et al., 2022). Increased iron accumulation, free-radical production, fatty-acid supply, and lipid peroxidation are crucial for the development of ferroptosis (Chen et al., 2021a). Key proteins in ferroptosis-related signaling pathways can be targeted by drugs or some small-molecule inhibitors.

Since the discovery of ferroptosis, many studies have confirmed that ferroptosis is related to the occurrence and development of many diseases, such as cancer, diabetes, and ischemia–reperfusion injury (Stockwell et al., 2017; Wei et al., 2020). According to the 10th edition of the International Diabetes Federation (IDF) diabetes atlas, diabetes is one of the fastest growing global health emergencies in the 21st century, and the number of people with diabetes worldwide is expected to reach 643 million by 2030 (Oka et al., 2021). Absolute or relative insufficiency of insulin secretion from pancreatic β-cells is the culprit of diabetes. Pancreatic islet cells are more sensitive to oxygen than other cells and have relatively weak antioxidant capacity. Pancreatic islets are prone to death when attacked by free radicals or when oxidative stress occurs in the islet microenvironment (Bottino et al., 2004; Wang and Wang, 2017). A number of small molecules have been found to improve β-cell function by inhibiting certain pathways of ferroptosis (Bannai et al., 1977; Zhou, 2020), which provides more possibilities for clinical treatment of diabetes. This article reviews the general mechanism of ferroptosis and the role of ferroptosis in the development of diabetes, thereby providing a comprehensive understanding for early diagnosis, treatment, and prognosis of diabetes.

## Molecular mechanisms of ferroptosis

As a new form of cell death, ferroptosis was originally discovered by the selective inhibition of erastin and RAS-selective lethal 3 (RSL3) (Yang and Stockwell, 2008). These two compounds are known to target RAS-mutant tumors (Dolma et al., 2003). Thereafter, detailed research has been performed on ferroptosis, demonstrating that compared with other forms of cell death, ferroptosis is a new, nonapoptotic programmed cell death (PCD).

### Inhibition of system Xc^−^–GSH–GPX4 activity promotes ferroptosis

System Xc^−^, a cystine/glutamate antiporter system, is an antiporter made up of two subunits, SLC7A11 (solute carrier family 7 member 11), which is the light chain of the subunit, and SLC3A2 (solute carrier family 3 member 2), which is the heavy chain of the subunit (Koppula et al., 2018). SLC7A11 is responsible for the main transport activity and is profoundly specific for cystine and glutamate, while SLC3A2 acts as a chaperonin. System Xc^−^ transports a molecule of glutamate out of the cell for every molecule of cystine delivered into the cell (Liu et al., 2021). Cystine is reduced to cysteine, which becomes the raw material for the synthesis of glutathione (GSH). Subsequently, GSH becomes a reducing cofactor for glutathione peroxidase, including glutathione peroxidase 4 (GPX4) and phospholipid hydroperoxide glutathione peroxidase (PHGPx).

GSH, which is mainly distributed in the cytoplasm, can scavenge reactive oxygen species (ROS) and play an important role in inhibiting ferroptosis (Wu et al., 2004). Inhibition of GSH synthesis leading to its depletion can lead to ferroptosis. l-glutamic acid, cysteine, and glycine are needed to synthesize GSH with energy provided by adenosine triphosphate (ATP). When erastin inhibits system Xc^−^, the source of cysteine is reduced and the amount of GSH synthesis decreases, which in turn leads to the accumulation of lipid peroxides, triggers protein and membrane damage, and initiates ferroptosis. Glutamate cysteine ligase (GCL) and GSH synthase (GS) are the rate-limiting enzymes in the GSH biosynthesis pathway. Inhibition of GCL and GS can lead to depletion of GSH and result in ferroptosis (Lu, 2009).

### GPX inactivation or depletion induces ferroptosis

GPX4 is the fourth member of the selenium-containing GPX family. In mammals, GPX4 is the only GPX family member with the ability to resist peroxide damage (Brigelius-Flohé and Maiorino, 2013). GPX4 reduces oxidative stress damage by converting the peroxy bonds of lipid peroxidation into hydroxyl groups so as to reduce lipid peroxides to lipid alcohols, despite the fact that these lipids have become a part of cell membranes or lipoproteins (Brigelius-Flohé and Maiorino, 2013; Seibt et al., 2019). As one of the mediators of oxidative stress, GPX4 is a core regulator of ferroptosis (Bersuker et al., 2019). PL-peroxidase activity of GPX4 is inhibited by RSL3 ((1S,3R)-RSL3), a ferroptosis inducer, by its binding to GPX4 selenocysteine (Sec) site (Yang et al., 2016). Boyi Gan’s team from the University of Texas MD Anderson Cancer Center found that uridine 5′-monophosphate (UMP) synthesis was significantly increased after treating cells with GPX4 inhibitors such as RSL3. This suggests that there may be a relationship between ferroptosis and pyrimidine nucleotide synthesis. It was next found that dihydroorotate dehydrogenase (DHODH) may be involved in the regulation of ferroptosis. Namely, the ferroptosis defense system located in mitochondria can regulate ferroptosis independently of the GSH pathway (Mao et al., 2021), and DHODH may serve as a novel target to restore cell death induced by ferroptosis.

### Depletion of reduced coenzyme Q10 (CoQ10) increases cellular susceptibility to ferroptosis

According to previous studies, ferroptosis suppressor protein 1 (FSP1) has a proapoptotic effect under certain conditions. FSP1 could translocate into the nucleus and trigger DNA degradation. However, recent studies have shown that FSP1 has an anti-ferroptosis mechanism parallel to the Cyst(e)ine-GSH-GPX4 axis. FSP1 reduces CoQ10 through NAD(P)H, which reduces lipid free radicals, thereby inhibiting lipid peroxidation and consequently inhibiting ferroptosis (Bersuker et al., 2019).

Unlike the Cyst(e)ine/GSH/GPX4 axis, which is dependent on the cysteine anti-transport system Xc^−^ and the cysteine-providing supersulfur pathway, the FSP1/CoQ10 axis is dependent on the mevalonate pathway. The mevalonate pathway involves the production of isopentenyl pyrophosphate (IPP), squalene (both IPP and squalene are precursors of CoQ10), CoQ10, and cholesterol (Moosmann and Behl, 2004). Inhibition of CoQ10 biosynthesis through the mevalonate pathway or inactivation of CoQ10 reductases such as FSP1 or DHODH can induce ferroptosis by increasing cellular susceptibility to ferroptosis in the absence of GPX4. Conversely, tetrahydrobiopterin (BH4) inhibits ferroptosis by converting phenylalanine to tyrosine to promote the synthesis of CoQ10 (Kraft et al., 2020).

### Induction of lipid ROS generation by means of iron and/or polyunsaturated fatty acid overload

As an iron-dependent form of cell death, ferroptosis requires high levels of iron. In the 1870s, Henry Fenton discovered that iron salts can react with peroxides to generate hydroxyl radicals, and proposed the famous fenton reaction (Fe^2+^ + HOOH →Fe^3+^ + OH^−^ + OH·). This reaction is also the basic principle of the lipid peroxidation step in ferroptosis. Namely, iron ions entering the cell undergo a Fenton reaction and peroxidize polyunsaturated fatty acids (PUFAs) to generate lipid peroxides (Li and Li, 2020). This process, regulated by three synthases, including acyl CoA synthase long-chain family member 4 (ACSL4), lysophosphatidylcholine acyltransferase 3 (LPCAT3), and lipoxygenase (LOX) (Doll et al., 2017), results in damage to the cell membrane structure and eventually leads to cell death. Therefore, iron metabolism is closely related to the occurrence of ferroptosis in cells, and it incorporates at least the following four aspects: intake, storage, utilization, and efflux (Chen et al., 2021b). Transferrin imports iron from the extracellular environment to intracellular space through recognition by transferrin receptor 1 (TfR1), while excess iron is bound to ferritin and transported outside of the cell under the action of ferroportin (FPN1), where the whole process normally maintains a dynamic balance (He et al., 2022).

## Ferroptosis in diabetes

**FIGURE 1:**
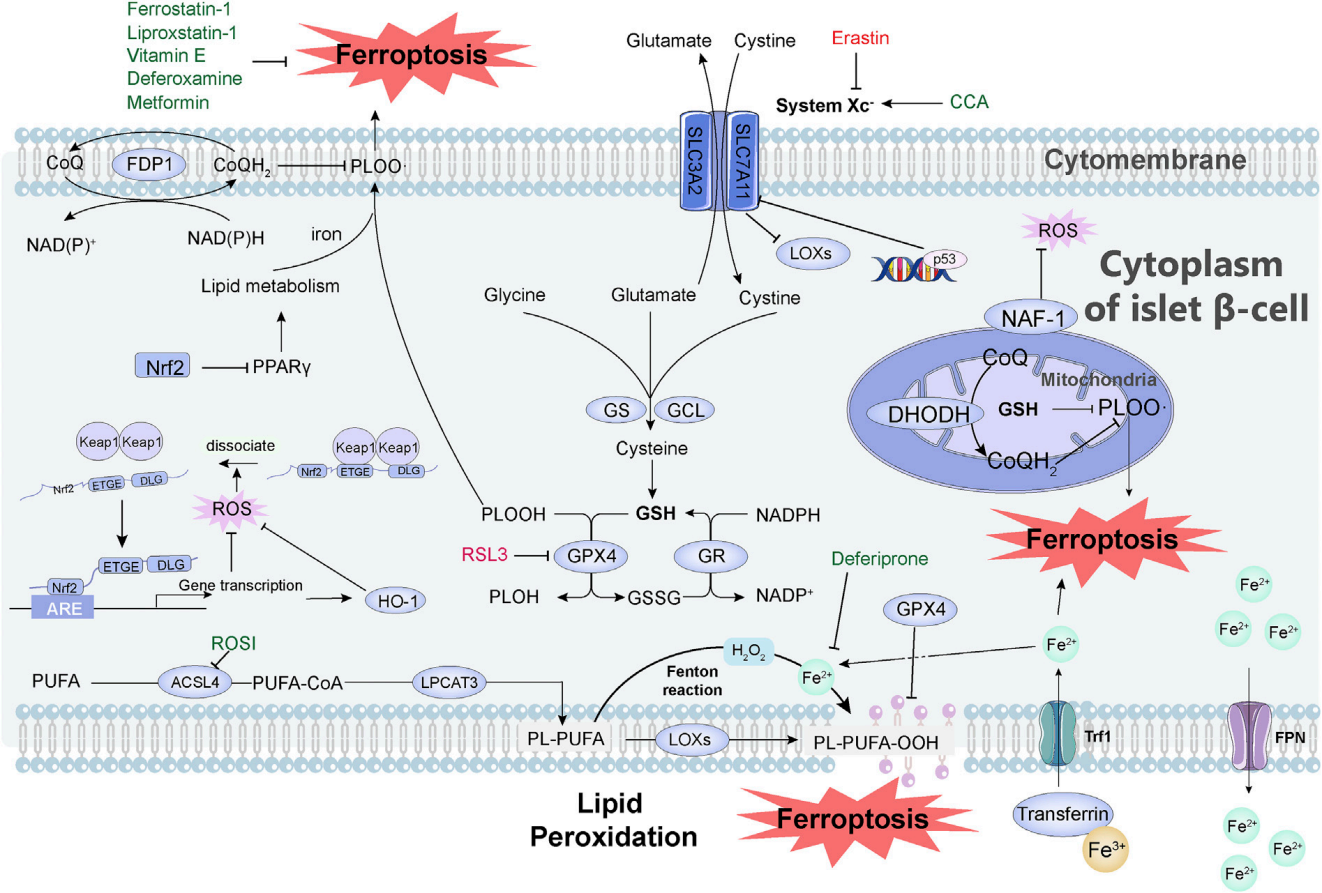
Mechanisms of how ferroptosis occurs and how it is inhibited in pancreatic islet cells. System Xc^-^ is a cystine/glutamate antiporter system consisting of SLC7A11 and SLC3A2. After cystine enters the cell, it is reduced to cysteine, which becomes the raw material for the synthesis of GSH, and GSH becomes the reducing cofactor of GPX4. Glutamate cysteine ligase (GCL) and GSH synthase (GS) are the rate-limiting enzymes in the GSH biosynthesis pathway. DHODH is involved in the regulation of ferroptosis independent of GSH pathway. FSP1 reduces CoQ through NAD(P)H, thereby reducing lipid free radicals and thereby inhibiting ferroptosis. Iron ions entering the cells undergo the Fenton reaction, which causes the peroxidation of PUFAs to generate lipid peroxides, resulting in the destruction of the cell membrane structure and the occurrence of ferroptosis. This peroxidation is regulated by three synthetases: ACSL4, LPCAT3, and LOX. Transferrin imports iron from the extracellular environment to the intracellular state through TfR1 recognition, and excess iron is stored in the form of bound ferritin and transported to cells under the action of FPN1. Transferrin imports Fe^3+^ (yellow ball) from the extracellular environment to the cytoplasm of islet β-cell through TfR1 recognition, and then Fe^3+^ is converted to Fe^2+^ (light green ball). The excess iron is stored in the form of bound ferritin and finally effluxed by FPN. Activation of p53 severely reduced the protein level of SLC7A11 and thus mediated ferroptosis. When the organism is under oxidative stress, Nrf2 dissociates from Keap1 and interacts with the ARE of target genes to maintain cellular redox homeostasis. The Nrf2-HO-1 pathway can modulate intracellular iron concentration to suppress ferroptosis. Nrf2 inhibits ferroptosis directly through the PPARγ pathway. Small molecules in green in the figure can inhibit ferroptosis, including: Vitamin E, Metformin, Deferoxamine, Liproxstatin-1, Ferrostatin-1, Deferiprone, CCA, ROSI. Small molecules in red can induce ferroptosis, including Erastin and RSL3. Abbreviation, System Xc^-^, a cystine/glutamate antiporter system; GSH, glutathione; GPX4, glutathione peroxidase; GCL, glutamate cysteine ligase; GS, GSH synthase; RSL3, RAS-selective lethal 3; DHODH, dihydroorotate dehydrogenase; FSP1, ferroptosis suppressor protein 1; CoQ, coenzyme Q; PUFAs, polyunsaturated fatty acids; ACSL4, acyl-CoA synthetase long-chain family member 4; LPCAT3, lysophosphatidylcholine acyltransferase 3; LOX, lipoxygenase; TfR1, transferrin receptor 1; FPN1, ferroportin; ARE, antioxidant response element; CCA, cryptochlorhydric acid; ROSI, rosiglitazone.

On the basis of the abovementioned molecular mechanisms of ferroptosis, multiple pathways are involved in the regulation of ferroptosis. We summarize the relationship between some pathways and diabetes, and reveal their potential applications in the diagnosis, treatment, and prognosis of diabetes (Figure 1). 

### Crucial role of p53 in ferroptosis-mediated diabetes

p53 is a tumor-suppressor gene in humans that causes cell cycle arrest, apoptosis, and/or senescence (Liu et al., 2019). One of the methods to activate the p53 protein signaling pathway is to disrupt the integrity of the DNA template (Liu and Gu, 2021). As shown by western blot (WB) analysis, the activation of p53 severely reduces the protein level of SLC7A11, and the flanking region of the SLC7A11 gene has a site that exactly matches the p53-binding sequence, indicating that the SLC7A11 gene is one of the downstream targets of p53 (Jiang et al., 2015; Liu and Gu, 2022). Gu’s team found that ALOX12 (arachidonate 12-lipoxygenase, 12S type) depletion had no significant effect on the expression of p53 and its downstream targets, but was able to rescue p53-mediated ferroptosis, indicating that ALOX12 is necessary for the p53-mediated ferroptosis pathway under ROS stress (Chu et al., 2019). They further constructed an ACSL4/GPX4 double-gene knockout (ACSL4^−/−^/GPX4^−/−^) human osteosarcoma cell line U2OS. The levels of p53 and its downstream targets were unaffected, but the cells underwent ferroptosis when exposed to TBH (tert-butyl hydroperoxide, mimicking ROS environment) and Nutlin (p53 activator), suggesting that p53 induces ferroptosis in a GPX4-independent manner (Chen et al., 2021c).

Minamino et al. (2009) demonstrated that upregulation of p53 in adipose tissue causes an inflammatory response that leads to insulin resistance. When β-cells were treated with free fatty acids (FFAs), the production of ROS increased and p53 was activated (Yuan et al., 2010). β-cell mitosis was reduced and their apoptosis was increased. Induction of the downstream microRNA miR34a sensitized β-cells to apoptosis and restrained insulin secretion. Akt plays an important role in promoting pancreatic β-cell survival by inhibiting the proapoptotic proteins such as glycogen synthase kinase 3α/β (GSK3α/β), Forkhead Box O1 (FoxO1), and p53 (Wrede et al., 2002; Lovis et al., 2008). Similar to Mdm2, ARF-BP1 acts as a ubiquitin ligase to control p53 stability and activity. The stability of p53 is tightly regulated by ARF-BP1 *in vivo*. Researchers constructed a mouse model with a specific deletion in pancreatic cells (*arf-bp1* ^*FL/Y*^ */RIP-cre*) and found that the mice died of severe diabetes as they matured; in contrast, when the p53 deletion was reversed (*p53* ^*LFL/FL*^ */arf-bp1* ^*FL/Y*^ */RIP-cre*), the mice lived longer (Kon et al., 2012). T-cell factor 7-like 2 (TCF7L2) is a key transcriptional effector of the Wnt/β-catenin signaling pathway, and leads to type 2 diabetes mainly by reducing β-cell survival and impairing insulin secretion. In glucose-stimulated INS-1 cells, TCF7L2 binds to gene promoters such as p53 and Fto. When TCF7L2 is silenced in INS-1 cells, p53INP1 protein expression is reduced, and apoptosis of INS-1 cells is decreased (Zhou et al., 2012). P53 is a key molecule for ferroptosis, which is linked to β-cell apoptosis and survival. Since p53 plays an important role in regulating physiological processes such as apoptosis, inflammation, and aging, targeting p53 in diabetes treatment may offer additional benefits.

### Nrf2 in ferroptosis-mediated diabetes

Nuclear factor erythroid 2-related factor 2 (NRF2) is a transcription factor that plays an important role in cellular antioxidant activity, regulates transcription of components of the GSH antioxidant systems, and is involved in phase I and phase II detoxification of exogenous and endogenous products, NADPH regeneration, and heme metabolism enzymes (Tonelli et al., 2018). As many of its downstream genes are associated with ferroptosis, Nrf2 is considered an important regulator of ferroptosis. When the organism is in a state of oxidative stress, Nrf2 dissociates from Keap1 and is transferred to the nucleus, where it interacts with antioxidant response elements (ARE) of target genes, activates transcriptional pathways, and maintains cellular redox homeostasis (Zhang, 2006). Selvakumar et al. found that the Nrf2-HO-1 pathway can regulate ferritin and thus intracellular iron concentration (Selvakumar et al., 2019). A protein–protein interaction network analysis revealed that Nrf2 mainly regulates ferroptosis by directly affecting the synthesis and function of Gpx4 and the PPARγ pathway (Song and Long, 2020).

Patients with type 2 diabetes mellitus commonly experience hyperglycemia, insulin resistance, inflammation, and dyslipidemia, all of which cause intracellular oxidative stress and inflammation (Karam et al., 2017). Flavin adenine dinucleotide hydroquinone form (FADH2) and nicotinamide adenine dinucleotide (NADH) generated by glucose or fatty acid oxidation undergo oxidative phosphorylation in the mitochondrial electron transport chain. When a large amount of oxidative phosphorylation occurs, the electron transport chain becomes congested, and the electrons return to complex I to generate ROS with oxygen (Fisher-Wellman and Neufer, 2012). Redox stress is a producer of diabetes-related tissue injury and causes serious complications.

Antioxidative enzymes are generally expressed at low levels in pancreatic endocrine cells, and treatment of islet cells with antioxidants rescues oxidative damage to β-cells in diabetic mice (Kaneto et al., 1999). The antioxidant ability of the Nrf2 pathway can maintain the body’s glucose level by saving the oxidative stress status of β-cells and maintaining insulin secretion. Cryptochlorhydric acid (CCA), a standout among the active components in mulberry leaf extract, has been shown to improve inflammation and insulin resistance (Tian et al., 2019). It inhibits ferroptosis by activating cystine/system x_c_^−^/Gpx4/Nrf2 and inhibiting NCOA4 in diabetes, thereby exerting a strong antidiabetic effect (Zhou, 2020).

### ACSL4 mediated-ferroptosis in β-cells

Acyl-CoA synthetase long-chain family member 4 (ACSL4) is an important enzyme in lipid metabolism, which catalyzes the reaction between long-chain fatty acids and coenzyme A to generate acyl-CoA. ACSL4 activates arachidonic acid to arachidonyl-CoA, which is further esterified to phospholipid (Liao et al., 2022). The oxidation of the endoplasmic reticulum–associated compartment involved in ferroptosis has been found to occur only on one class of phospholipids [phosphatidylethanolamines (PEs)] and targets two fatty acids [arachidonoyl (AA) and adrenoyl (AdA)] (Kagan et al., 2017). Doll et al. found that exogenous arachidonic acid enhanced RSL3-induced ferroptosis, and ACSL4 was a key enzyme in arachidonic acid–induced ferroptosis in synergy with IFNγ (Doll et al., 2017; Liao et al., 2022). The expression of ACSL4 can reflect the sensitivity of cells to ferroptosis (Yuan et al., 2016).

The ACSL4 protein exists in the β-cells of human and rat islets, and its distribution site suggests that ACSL4 is involved in insulin secretion by modifying fatty acids in insulin secretion granules and mitochondria (Ansari et al., 2017). Upregulation of ACSL4 expression was observed in mice fed a high-fat diet, and when ACSL4 was specifically knocked out in adipocytes in mice fed a high-fat diet, the mice were protected from insulin resistance (Killion et al., 2018). Some studies have found that when the ACSL4 inhibitor rosiglitazone (ROSI) is used in diabetic nephropathy mice, it may inhibit the inflammatory response, thereby inhibiting ferroptosis, and finally improving the damage of renal tubular cells in a high-glucose environment (Wang et al., 2020), which also provides some ideas for ACSL4 as a potential therapeutic target for diabetes.

### WFS-T2 in β-cells

WFS-T2 encodes the protein NAF-1. NAF-1 is a member of the NEET protein family, a highly conserved [2Fe-2S] protein. NAF-1 localizes to mitochondria, the endoplasmic reticulum (ER), and the mitochondria-associated membrane connecting these organelles, and its unique [2Fe-2S] cluster structure makes NAF-1 essential for autophagy, ferroptosis, redox, and oxidative stress. It plays a key regulatory role in the process of cell proliferation (Nechushtai et al., 2020). Researchers built an NAF-1 knockout INS-1 cell model, and the cells developed ferroptosis-like features such as enhanced lipid peroxidation, mitochondrial atrophy, and GPX4 expression. Insulin secretion was impaired in NFA-1 knockout INS-1 cells. When this cell model was treated with the iron chelator deferiprone, the GSH precursor N-acetyl cysteine, and the ferroptosis inhibitor ferrostatin-1, the levels of ROS generated by the cells were reduced, the mitochondrial and endoplasmic reticulum structures were improved, and cellular insulin secretion function was repaired (Agrawal et al., 2018; Ommati et al., 2021).

## Conclusion and perspectives

This article reviews the mechanism of ferroptosis and the role of ferroptosis in diabetes. At present, diabetes is one of the most prevalent diseases in the world, and its complex pathogenesis and systemic complications make it a very difficult problem. As a new form of PCD, ferroptosis has different characteristics from other cell deaths, and has shown great potential in the diagnosis, treatment, and prognosis of diabetes. Four mechanisms for the induction of ferroptosis have been identified: (Dixon et al., 2012): inhibition of the system Xc^−^, (Chen et al., 2021a) inhibition/degradation/inactivation of GPX4, (Lei et al., 2022) depletion of reduced CoQ10, and (Stockwell et al., 2017) lipid peroxidation *via* iron or polyunsaturated fatty acid overload.

Given the association between diabetes and ferroptosis, starting from the key targets of ferroptosis might improve diabetes. Improvements in insulin secretion and insulin sensitivity along with better control of blood glucose have been observed after reducing iron storage levels in the body (Houschyar et al., 2012). Although serum ferritin levels can be for early diagnosis of type 2 diabetes mellitus and gestational diabetes mellitus (Wang et al., 2018), the use of serum ferritin levels to calculate iron stores in the body appears unreliable because ferritin is also elevated in other diseases such as cancer and liver disease (Wang et al., 2010). In the treatment of diabetes, many specific ferroptosis inhibitors have been identified, such as ferrostatin-1, liproxstatin-1, vitamin E, and deferoxamine (Ju et al., 2021). Although many drugs are still in the preclinical stage, some drugs that have been marketed (such as metformin) have been shown to inhibit ferroptosis and may be beneficial for diabetes and its complications (Ma et al., 2021). This can provide ideas for the application of ferroptosis in diabetes treatment.

However, there are still some unanswered questions about the occurrence and development of ferroptosis in diabetes. For example, as there are no markers of ferroptosis *in vivo*, we cannot be sure whether ferroptosis occurs during cell growth and differentiation. In addition, ferroptosis is caused by phospholipid peroxidation, and ROS are also closely related to ferroptosis. Considering the oxygen demand of islet cells, it is unclear whether the ferroptosis process is dependent on the concentration of oxygen. In conclusion, although ferroptosis has not been thoroughly studied and its molecular mechanism in diabetes and diabetic complications needs to be further explored, ferroptosis is a potential target for the therapeutics of diabetes.
